# Acute Kidney Injury in Critically Ill Children: Prevalence, Progression, Recovery Mortality, and Impact of Severity

**DOI:** 10.3390/jcm14030886

**Published:** 2025-01-29

**Authors:** Mohammed Naeem, Seham Alarishi, Fatmah Othman, Mohammed Alfurayh, Hamad Alkhalaf

**Affiliations:** 1 Department of General Pediatrics, Ministry of the National Guard–Health Affairs, Riyadh P.O. Box 11426, Saudi Arabia; naeemm@mngha.med.sa (M.N.); alfurayhmo@ngha.med.sa (M.A.); 2 College of Medicine, King Saud bin Abdulaziz University for Health Sciences, Riyadh P.O. Box 11481, Saudi Arabia; 3 King Abdullah International Medical Research Center, Riyadh P.O. Box 3660, Saudi Arabia; 4 Department of Pediatric ICU, King Fahad Central Hospital, Jizan P.O. Box 82666, Saudi Arabia; dr.seham1407@hotmail.com; 5 Epidemiology and Biostatistics Department, College of Public Health and Health Informatics, King Saud bin Abdulaziz University for Health Science, Riyadh P.O. Box 11481, Saudi Arabia; othmanf@ksau-hs.edu.sa

**Keywords:** acute kidney injury, mortality, PICU, creatinine, morbidity

## Abstract

**Introduction**: Acute kidney injury (AKI) among the pediatric population is considered a risk factor for mortality and morbidities during pediatric intensive care unit (PICU) admission. The association between AKI and increased mortality risk and length of stay (LOS) is still inconclusive. This retrospective cohort study aimed to evaluate the impact of AKI severity upon critical management and clinical parameters with an evaluation of severity progression among AKI patients admitted to the PICU at a tertiary care hospital. **Methods**: AKI, defined with the Kidney Disease Improving Global Outcomes (KDIGO) classification, was determined based on serum creatinine and urine output with respect to the patient’s baseline value. The following outcomes were examined: mortality, mechanical ventilation use, use of non-invasive ventilation, recovery at discharge, and LOS in the hospital and PICU stratified by type of AKI upon admission. Medical records of the 165 included patients were reviewed for clinical data and study outcomes. **Results**: The median age of the patients was 7 years (IQR 1.5–10 years), and 58% were boys; 78 (47.2%) had stage 1 AKI, 49 (29.71%) had stage 2 AKI, and 38 (23%) had stage 3 AKI at admission. The mortality rate was 26%. The median LOS in the PICU was statistically significant between AKI stages, with a higher median LOS among patients with AKI stage 3 at admission. Using the maximum KDIGO stage, there was no association between AKI and mortality (adjusted OR 1.91, 95% CI 0.05), whereas for the mechanical ventilation outcome, the adjusted OR was 1.84 (95% CI 0.42–8.1). **Conclusions**: The severity of AKI is not associated solely with mortality and clinical outcomes as the numbers of comorbidities and organ failures contribute to mortality. However, improving awareness of AKI and understanding the disease progression course would reduce acute and long-term morbidity and mortality.

## 1. Introduction

Acute kidney injury (AKI) is a clinical condition characterized by an abrupt decrease in renal function that causes abnormalities in fluid homeostasis, urine output, and serum electrolytes. It affects millions of children across the globe every year [1].

The two standardized and validated tools most frequently used to identify and characterize AKI are Kidney Disease Improving Global Outcomes (KDIGO) and Pediatric Risk, Injury, Failure, Loss, End-Stage Renal Disease (pRIFLE) [2]. KDIGO utilizes two measurements: (1) serum creatinine and (2) urine output and defines a patient as having AKI if they have one or more of the following: (a) an increase in serum creatinine by ≥0.3 mg/dL from baseline (≥26.5 mcmol/L) within 48 h; (b) an increase in serum creatinine to ≥1.5 times baseline within the prior seven days; or (c) urine output ≤ 0.5 mL/kg/h for six hours [3]. AKI can result from prerenal, intrinsic renal, and postrenal causes involving one or multiple factors, including, but not limited to, hemodynamics, hypoxemia, immune deregulation, and medications.

This study aimed to explore AKI by (1) evaluating the prevalence of AKI and relevant specific clinical and laboratory data of the identified subjects; (2) evaluating AKI progression; (3) evaluating the impact of the degree of AKI severity upon critical management and clinical parameters, including hospital and PICU length of stay (LOS), utilization of mechanical ventilation, and vasopressor treatment; and (4) identifying the impact of AKI severity upon renal function recovery and mortality.

## 2. Materials and Methods

### 2.1. Study Design and Setting

A retrospective cohort study was carried out at the PICU in the tertiary care hospital King Abdullah Specialized Children’s Hospital (KASCH), Riyadh. The hospital serves eligible Saudi National Guard soldiers, employees, and their families, with the PICU having a capacity of 40 beds.

### 2.2. Study Population, Variables, Data Collection, and Definitions

Pediatric patients (aged 1 day to less than 14 years) who were admitted to the PICU over three consecutive years with a diagnosis of AKI were included in this study. AKI was defined with the KDIGO classification and determined based on serum creatinine and urine output relative to the patient’s baseline value [2]. AKI patients were classified as stage 1 if there was an increase in serum creatinine of more than 26.5 mmol, an elevation in serum creatinine of 1.5–1.9 times the patient’s baseline, or diuresis < 0.5 mL/kg/h for 6 to 12 h; as AKI stage 2 if there was an elevation of serum creatinine of 2–2.9 times the patient’s baseline or diuresis < 0.5 mL/kg/h for >12 h; and as stage 3 if there was an elevation of serum creatinine ≥ 3 times, an acute increase > 4 mg/dL, initiation of Continuous Renal Replacement Therapy (CRRT), a decrease in eGFR by 35 mL/min/1.73 m^2^, diuresis < 0.3 mL/kg/h for >24 h, or anuria > 12 h. From this population, children with known structural or congenital renal abnormalities, a history of renal transplant, or who were under 28 days of age were excluded. Recovery was defined as serum creatinine having reverted to the baseline level.

Based on previous studies, the incidence of AKI was estimated to be around 18% in PICU patients. Therefore, a minimum sample of 114 patients was required, assuming absolute precision d = 0.07 and z score = 1.96.

The medical records of the included patients were reviewed for demographic and clinical data. Data on comorbidities and organ failure were extracted for each patient. The levels of creatinine, urine output, and other laboratory tests were extracted from the medical files upon PICU admission and at regular intervals of 3, 5, 7, 10, 14, 21, 30, 60, and 90 days thereafter, depending on the duration of each child’s stay in the unit. The study variables—mortality, hospital LOS, PICU LOS, need for renal replacement therapy, recovery from AKI, and use of mechanical ventilation—were assessed until discharge.

Institutional Review Board approval was obtained from King Abdullah International Medical Research Center (approval #RC19-160-R) prior to this study.

### 2.3. Statistical Analysis

For the demographic data and clinical parameters, descriptive statistics were used to analyze the variables within the sample. Continuous variables were presented as means and standard deviations or medians with interquartile ranges based on the distribution. The Shapiro–Wilk test was used to test for normality. Categorical variables were described using frequencies and percentages.

Patients with a diagnosis of AKI were classified into KDIGO categories (stages 1, 2, and 3) as described at admission and presented as frequencies and proportions. In addition, the maximum KDIGO stage during the PICU stay was estimated for each patient. The progression of AKI, in terms of transitioning to a lower or higher stage, was estimated for all patients for up to three days after admission. The same analysis was conducted for patients still in the PICU at 5 and 14 days.

The following outcomes were examined among AKI patients: mortality, mechanical ventilation use, use of non-invasive ventilation, recovery at discharge, and hospital and PICU lengths of stay (LOS), stratified by type of AKI upon admission. The chi-squared test (or Fisher’s exact test, as appropriate) was used to examine the association between categorical variables, whereas the Kruskal–Wallis H test was used to determine if there were statistically significant differences between KDIGO stages and hospital or PICU LOS.

We carried out a logistic regression analysis to examine the association between AKI stage (measured as maximum KDIGO stage during the PICU stay) and the study outcomes. Odds ratios (ORs) and 95% confidence intervals were calculated. We used survival analysis and the Kaplan–Meier method to determine the risk of prolonged LOS in the PICU based on AKI stages. Statistical significance was determined as a *p*-value of 0.05. All analyses were performed using STATA 15.0 (College Station, TX, USA).

## 3. Results

In total, 1722 patients were admitted to the PICU during the study period, of whom 342 were excluded for not having baseline creatinine data on admission. Among the remaining 1380 patients, 372 were found to have AKI. Among those, 165 (44%) patients had AKI at the time of admission and were included in the analysis.

### 3.1. Description of the Clinical and Laboratory Findings

Patient demographic data and clinical characteristics are shown in Table 1. The median age of the patients was 7 years (IQR 1.5–10 years), and 58% were boys. Around 54% of the subjects had one or more co-existing comorbidity or comorbidities at admission, and 66% had at least two system organ failures (Table 1). The highest serum creatinine was 102 (IQR 75–134) mg/dL. The electrolyte parameters among the admitted patients at admission (i.e., highest and lowest levels) are reported in Table S1, along with the chemical and coagulation profiles (Table S2).

### 3.2. Impact of Severity of AKI Age, Long-Term Comorbidities, and Acute Multiple Organ Dysfunction Syndrome

**Figure 1:**
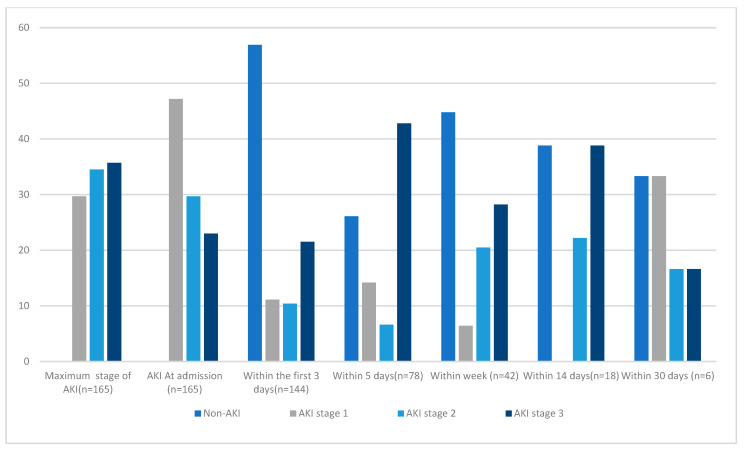
Daily frequency of the stage of AKI among patients admitted to the ICU according to the KDIGO stage. Note: the number of patients changes during the LOS in the PICU.

According to the KDIGO definition, 78 (47.2%) had stage 1 AKI, 49 (29.71%) had stage 2 AKI, and 38 (23%) had stage 3 AKI at admission. The daily frequency of AKI and the maximum stage of AKI during the PICU stay are shown in Figure 1. There were significant associations between age, number of co-existing comorbidities, and number of organ failures with the presence of severe AKI (Table 2). In regard to age, children under 3 years old had a higher percentage of AKI stage 3 than children over 3 years old, namely 34% for children under 1 year of age and 31% for children aged between 1 and 3 years old. In terms of comorbidities, the percentage of patients with AKI stage 3 who had fewer than two comorbidities was higher than that of patients with two or more comorbidities (68% vs. 31%, respectively, *p*-value = 0.050). We noticed a clinically significantly higher number of stage 3 AKI patients with two or more organ dysfunctions than of children who had fewer than two system organ failures (24% vs. 26%, *p*-value = 0.005). A similar trend was noticed in terms of vasopressor support and use of antibiotics (*p*-values = 0.005 and 0.001, respectively) (Table 2).

### 3.3. AKI Progression

In terms of progression in the first three days, 44% of stage 1 AKI patients recovered completely, and about 81% and 48% of stage 2 and stage 3 AKI patients, respectively, had stepped down. It was observed that the AKI progression trend was more inclined to be static after three days of admission (Table 3). Most of the patients who were still in the PICU on the fifth day after admission maintained the same stage of AKI (57%, 43%, and 66% of the patients with stage 1, stage 2, and stage 3 AKI, respectively). The progression of the patients during the first two weeks in terms of recovery to the lower AKI stage or progressing to the more severe AKI stage is shown in Table 3.

### 3.4. Impact of Severity of AKI upon Key Mortality, Clinical Parameters, and Management

The mortality rate was 26% among the 165 patients with AKI: 33% mortality in patients with stage 1 AKI at admission, 18% with stage 2 AKI, and 21% with stage 3 AKI (*p*-value = 0.136; Table 3). For recovery at discharge, 67% of the total population had complete renal recovery without differences between AKI stages.

**Figure 2:**
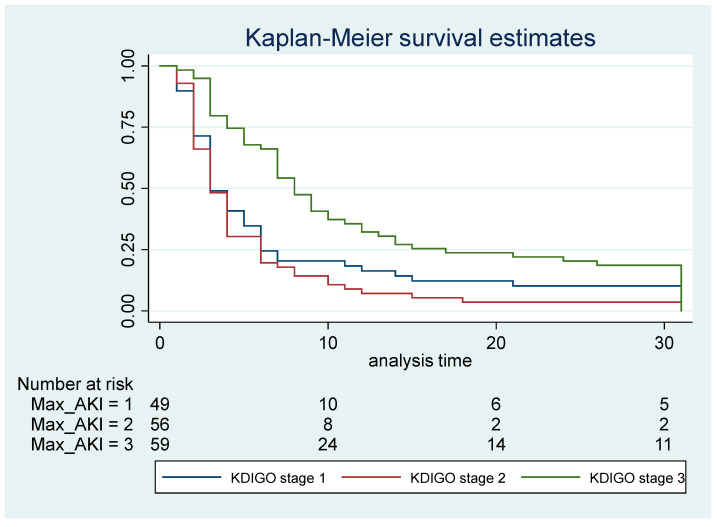
Median length of stay in the PICU stratified by KDIGO AKI stage at admission.

The median LOS in the PICU was statistically significant among AKI stages, with a higher median LOS in the PICU among patients with AKI stage 3 at admission (Figure 2). This was similar to the LOS in hospital. Patients with AKI stage 3 (63%) were more likely to be placed on mechanical ventilators than those with AKI stage 1 (56%) or stage 2 (36%), *p*-value = 0.029 (Table 4).

### 3.5. Impact of Stages upon Outcomes

Using the maximum KDIGO stage, the unadjusted OR for mortality among AKI stage 2 compared to stage 1 was 2.86 (95% CI 0.94–8.64), and this remained insignificant after adjustment for confounders (Table 5). Patients with AKI stage 3 had a higher risk of mortality than those with AKI stage 1 (OR 6.01, 95% CI 2.08–17.4); however, this risk was reduced after adjustment for age, gender, presence of comorbidity, organ failure, receiving renal replacement therapy, and receiving furosemide infusion (adjusted OR 1.91 95% CI 0.50–7.25).

For the mechanical ventilation outcome, patients with AKI stage 3 were seven times more likely to be placed on mechanical ventilation than those with AKI stage 1 (OR 7.28, 95% CI 3.10–17.1), and after adjustment for confounders, the OR was 1.84 (95% CI 0.42–8.11).

For recovery at discharge, patients at AKI stage 3 during the PICU stay were 36% less likely to have complete renal recovery than those with AKI stage 1 (95% CI 0.13–1.02) after adjustment for confounders (Table 5).

## 4. Discussion

Among 1380 patients, 372 were found to have AKI, a 25% prevalence that is consistent with other studies [4,5]. Of the total patients with AKI, this study evaluated those who presented with AKI at admission (165, 44%). Not surprisingly, this study found outcomes similar to those reported in previous investigations regarding patient demographics, LOS, mortality, use of critical modalities such as mechanical ventilation, and the relationship of outcomes to AKI severity in a dose-dependent manner.

Regarding recovery and progression, ongoing investigations are being conducted in the pediatric population, and notable studies are already available on AKI in adults [6,7,8]. Our study found that 67% of pediatric AKI cases had complete renal recovery. Regarding recovery at discharge, patients at AKI stage 3 during the PICU stay were 36% less likely to have complete renal recovery than those with AKI stage 1 (95% CI 0.13–1.02) after adjustment for confounders [9,10]. Overall renal recovery, when defined as a serum creatinine < 1.5× baseline (no longer meeting stage 1 AKI criteria), was 67%. Not surprisingly, this study found a dose-dependent effect, with recovery being less common as AKI severity increased; renal recovery occurred in 88%, 58%, and 44% of adults with stage 1, stage 2, and stage 3 AKI, respectively [11]. Hessey et al. examined 2033 pediatric ICU admissions and found that the likelihood of recovery was highly dependent on how it was defined [9]. For example, 92.5% of patients with AKI recovered function when it was defined as a discharge creatinine < 1.5× baseline, but recovery only occurred in 75.9% when it was defined as a discharge creatinine < 1.15× baseline [12].

Regarding complete AKI recovery, we are aware that a patient may develop chronic renal disease even if their creatinine level is normal at discharge. AKI survivors have a significant prevalence of CKD, hypertension, and proteinuria, according to multiple observational studies [13,14]. Moreover, all of our patients whose creatinine levels normalized have been followed in the nephrology clinic, ensuring they receive appropriate ongoing care. For those with persistent abnormal kidney function, we have also provided the necessary management and follow-up.

Regarding progression, most patients with stage 1, 2, or 3 AKI showed recovery within the first three days of admission to the PICU. Afterward, the predominant modes of progression were static or slow during the entire 30-day follow-up. Variability in progression is multifactorial, with early management being one significant factor (Table 3). In fact, the recovery ratio is significantly affected by changes in the definition of recovery [10,12]. In the adult population, Long et al. found a 67% recovery in 10,419 subjects with AKI; similar to our study, the recovery was highest in stage 1, followed by stages 2 and 3, at 88%, 58%, and 44%, respectively [10]. In the pediatric population, researchers evaluating recovery in 2033 pediatric patients with AKI found that recovery assessment is complex and that the definition of recovery significantly impacts the recovery rate [12].

In our study, the length of hospital stay was highest in patients with AKI stage 3, with a median of 8 days, compared to 3 days for stage 2 and 5 days for stage 1. Similarly, the length of PICU admission was six days in AKI stage 3, two days in stage 2, and three days in stage 1.

The length of hospital stay (LOS) in pediatric patients increases with the severity of acute kidney injury (AKI). Studies have shown that children with AKI experience longer hospitalizations than those without AKI, and this duration extends further with higher AKI stages [15].

For instance, research indicates that the average LOS for pediatric patients without AKI is approximately 5.76 days. In the presence of AKI, this average increases to 8.4 days, reflecting a significant extension in hospitalization [16].

Furthermore, the LOS tends to increase progressively with the severity of AKI. A study analyzing data from 20 hospitals in China found that the average LOS and hospitalization costs increased progressively with AKI stage.

These findings underscore the importance of early detection and management of AKI in pediatric patients to potentially reduce hospitalization duration and associated healthcare costs [15].

Adequate fluid provision is an important component in the care of children with AKI [17].

To guarantee proper end-organ perfusion, particularly of the kidneys, early volume replacement is crucial in the course of critical illness. Fluid management techniques to reduce unnecessary fluid overload (FO) can directly affect renal function by halting the progression of edema and abdominal pressure after a patient has received sufficient resuscitation.

Although the effects of FO on the lungs have been widely studied, FO can also impair kidney function by negatively affecting encapsulated organs like the kidneys when interstitial edema and elevated venous pressure occur [17].

In our study, seven patients underwent fluid restriction during their hospital course; four had stage 3 AKI, and three had stage 1 AKI.

### Limitations

Although the current study sheds light on the progression of AKI, certain limitations must be considered when interpreting the findings. The effect of confounders cannot be ruled out due to the nature of this study. However, a comprehensive list of comorbidities and laboratory findings was extracted from the medical files and included in the adjustment estimates. Second, this study was carried out in a single center, which limits the generalizability of the findings.

## 5. Conclusions

The guidelines about the prevention, detection, and management of AKI in children have become clearer in recent years; however, outcomes such as progression in terms of recovery are not as well studied in children as in adults. Hence, larger-scale RCTs should be conducted to complement this study’s findings.

## Figures and Tables

**Table 1:** Demographic data and clinical characteristics of patients with AKI who were admitted to the PICU.

Variable	All Patients *N* = 165
Age group	
<1 year	30 (18.1)
From 1 year to 3 years	41 (24.8)
More than 3 years up to 10 years	45 (27.2)
More than 10 years	49 (29.7)
Gender	
Female	68 (41.2)
Male	97 (58.7)
BMI (mean ± SD)	15 ± 4
Co-existing comorbidity	
Any comorbidity	90 (54.5)
Organ-specific	
Respiratory	31 (18.7)
Cardiovascular	23 (13.9)
Central nervous system	24 (14.5)
Liver disease	7 (4.2)
Other	63 (38.1)
Organ failure	
Renal failure	135 (81.8)
Respiratory	68 (41.2)
Cardiovascular	75 (45.1)
Liver failure	68 (41.2)
Central nervous system	65 (39.3)
Other cause of organ failure	3 (1.8)
Patients with two or more organ failure	99 (60.0)
Primary diagnosis at PICU admission	
Respiratory	35 (21.2)
Central nervous system	32 (19.3)
Cardiovascular	13 (7.8)
Renal	7 (4.2)
Coagulation	2 (1.2)
Liver	5 (3.0)
Other	71 (43.1)

PICU: pediatric intensive care unit.

**Table 2:** The clinical data and medication received by the patients with AKI diagnosis based on the KDIGO stage criteria (stages 1, 2, and 3) on admission to the PICU.

Variable	All	AKI Staging at Admission			*p*-Value
		Stage 1	Stage 2	Stage 3	
Age group					0.017
<1 year	30 (18.1)	8 (10.2)	9 (18.3)	13 (34.2)	
From 1 year to 3 years	41 (24.8)	20 (25.6)	9 (18.3)	12 (31.58)	
More than 3 years up to 10 years	45 (27.2)	27 (34.6)	13 (26.5)	5 (13.1)	
More than 10 years	49 (29.7)	23 (29.4)	18 (36.7)	8 (21.1)	
Comorbidities					0.050
Below 2	124 (75.1)	55 (70.5)	43 (86.7)	26 (68.4)	
2 or more	41 (24.8)	23 (29.4)	6 (12.2)	12 (31.5)	
Number of organ failure					0.005
Below 2	66 (40.0)	29 (37.1)	28 (57.1)	9 (23.6)	
2 or more	99 (60.0)	49 (62.8)	21 (42.8)	29 (76.3)	
Medication used					
Midazolam	62 (37.5)	33 (42.3)	13 (26.5)	16 (42.1)	0.162
Fentanyl	74 (44.8)	38 (48.7)	13 (26.5)	23 (60.5)	0.004
Morphine	6 (3.6)	4 (5.1)	1 (2.0)	1 (2.6)	0.867
Pancuronium	23 (13.9)	17 (21.7)	2 (4.1)	4 (10.5)	0.014
Cisatracurium	23 (13.9)	18 (23.1)	1 (2.0)	4 (10.5)	0.002
Inotrops	72 (43.6)	40 (51.2)	12 (24.4)	20 (52.6)	0.005
Epinephrin	64 (38.7)	39 (50.0)	8 (16.3)	17 (44.7)	0.001
Norepinephrin	30 (18.1)	17 (21.7)	6 (12.2)	7 (18.4)	0.442
Dopamine	41 (24.8)	28 (35.9)	6 (12.2)	7 (18.4)	0.006
Dobutamine	6 (3.6)	4 (5.1)	-	2 (5.2)	0.232
Milrinone	29 (17.5)	14 (17.9)	6 (12.2)	9 (23.6)	0.372
Vasopressin	8 (4.8)	4 (5.1)	1 (2.0)	3 (7.8)	0.44
Antibiotics	114 (69.1)	59 (75.6)	24 (48.9)	31 (81.5)	0.001

**Table 3:** Progression of AKI at 3, 5, and 7 days after admission to the PICU.

AKI Progression	AKI KDIGO Stage 1	AKI KDIGO Stage 2	AKI KDIGO Stage 3
During the first 3 days (*n* = 144)			
Stepped down	30 (44.1)	35 (81.4)	16 (48.4)
Progressed	19 (27.9)	4 (9.3)	-
Stayed the same	19 (27.9)	4 (9.3)	17 (51.5)
From the first 3 days to 5 days (*n* = 78)			
Stepped down	7 (18.4)	7 (43.7)	8 (33.3)
Progressed	9 (23.6)	2 (12.5)	-
Stayed the same	22 (57.8)	7 (43.7)	16 (66.6)
From 5 days to 14 days (*n* = 42)			
Stepped down	7 (33.3)	2 (33.3)	3 (80.0)
Progressed	4 (19.1)	-	-
Stayed the same	10 (47.6)	4 (66.6)	3 (20.0)

**Table 4:** Outcomes in patients with AKI diagnosis based on the KDIGO stage criteria (stage 1, 2, and 3) on admission.

Outcomes	Total (*n* = 165)	AKI KDIGO Stage 1 (*n* = 78)	AKI KDIGO Stage 2 (*n* = 49)	AKI KDIGO Stage 3 (*n* = 38)	*p*-Value
Mortality	43 (26.1)	26 (33.3)	9 (18.3)	8 (21.1)	0.136
LOS in PICU in days (median, IQR)	3 (1–8)	3 (1–9)	2 (1–5)	6 (2–16)	<0.001
LOS in hospital in days (median, IQR)	4 (3–10)	5 (3–12)	3 (2–4)	8 (4–24)	<0.001
Use of mechanical ventilation	86 (52.1)	44 (56.4)	18 (36.7)	24 (63.1)	0.029
Days in mechanical ventilation (median, IQR)	5 (2–9)	4 (1–8)	4 (3–8)	7 (3–13)	0.146
High-frequency mode	33 (38.3)	21 (47.7)	3 (16.6)	9 (37.5)	0.065
Use of non-invasive ventilation	15 (9.1)	6 (7.8)	3 (6.2)	6 (15.7)	0.302
Recovery at discharge (as two categories)					0.408
Complete recovery	112 (67.8)	49 (62.8)	36 (73.4)	27 (71.1)	
Not recovered	53 (32.1)	29 (37.1)	13 (26.5)	11 (28.9)	
Receiving renal replacement therapy	17 (10.3)	8 (10.2)	1 (2.0)	8 (21.1)	0.014
Furosemide infusion	31 (18.7)	18 (23.1)	3 (6.1)	10 (26.3)	0.015
Fluid restriction	7 (4.2)	3 (3.8)	0	4 (10.5)	0.032

IQR: interquartile range. LOS: length of stay. PICU: pediatric intensive care unit.

**Table 5:** The risk of study outcomes in relation to the AKI diagnosis based on the maximum KDIGO stage criteria (stage 1, 2, and 3).

	Using the Maximum KDIGO Stage			
	Unadjusted OR (95%CI)	*p*-Value	Adjusted OR (95%CI) *	*p*-Value
Mortality				
AKI stage 1	ref		ref	
AKI stage 2	2.86 (0.94–8.64)	0.062	3.20 (0.78–13.11)	0.105
AKI stage 3	6.03 (2.08–17.43)	0.001	1.91 (0.50–7.25)	0.337
Use of mechanical ventilation				
AKI stage 1	ref		ref	
AKI stage 2	1.90 (0.85–4.23)	0.116	1.52 (0.39–5.84)	0.541
AKI stage 3	7.28 (3.10–17.10)	<0.001	1.84 (0.42–8.11)	0.416
Use of non-invasive ventilation				
AKI stage 1	ref		ref	
AKI stage 2	1.12 (0.23–5.31)	0.879	1.24 (10.71–8.65)	0.826
AKI stage 3	2.30 (0.57–9.20)	0.239	0.34 (0.04–2.36)	0.278
Recovery at discharge				
AKI stage 1	ref		ref	
AKI stage 2	0.45 (0.17–1.18)	0.107	0.46 (0.16–1.33)	0.157
AKI stage 3	0.21 (0.086–0.53)	0.001	0.36 (0.13–1.02)	0.051

Separate models for each of the following outcomes (mortality, use of mechanical ventilation, use of non-invasive ventilation, and recovery at discharge). * Adjusted for age, gender, presence of comorbidity (no comorbidity, one or more comorbidities), organ failure (categorized into fewer than two organ failures or two or more organ failures), receiving renal replacement therapy, and receiving furosemide infusion. KDIGO: Kidney Disease Improving Global Outcomes. AKI: Acute kidney injury.

## Data Availability

The original contributions presented in this study are included in the article/Supplementary Material. Further inquiries can be directed to the corresponding author.

## Supplementary Materials

The following supporting information can be downloaded at: https://www.mdpi.com/article/10.3390/jcm14030886/s1, Table S1: Electrolyte parameters among patients with AKI diagnosis; Table S2: Chemical and coagulation profile of patients with AKI diagnosis.
