# Supplementary material for: Acute Kidney Injury in Critically Ill Children: Prevalence, Progression, Recovery Mortality, and Impact of Severity
Source: J Clin Med. 2025 Jan 29;14(3):886. doi: 10.3390/jcm14030886 (PMC11818388; doi:10.3390/jcm14030886)
Supplement: Supplementary file 1 [file jcm-14-00886-s001.zip › jcm-3388042-supplementary.pdf]

Table S1: Electrolyte parameters among patients with AKI diagnosis

**Variable**	**All patients**<br>**N=165**
Serum creatinine at admission	0.85(0.71–1.29) mg/dL
Serum creatinine highest	1.15(0.71–1.25) mg/dL
Serum creatinine lowest	0.5(0.4–0.6) mg/dL
BUN at admission	17.9(11.7–25.2) mg/dL
BUN highest	23.5(15.7–42) mg/dL
BUN lowest	7(4.2–10.4) mg/dL
Sodium at admission	137(133–141) meq/L
Sodium highest	144(139–152) meq/L
Sodium lowest	133(130–139) meq/L
Potassium at admission	4.3(3.7–5) meq/L
Potassium highest	5(4.7–5.6) meq/L
Potassium lowest	3(2.8–3.5) meq/L
Glucose at admission	155(104–429) mg/dL
Glucose highest	342(198–504) mg/dL
Glucose lowest	79(70–106) mg/dL
Bicarbonate at admission	16(6–19) meq/L
Bicarbonate highest	24(21–28) meq/L
Bicarbonate lowest	11(5–16) meq/L
Calcium at admission	8.5(8.1–8.6) mg/dL
Calcium highest	9.2(8.8–9.6) mg/dL
Calcium lowest	7.6(6.4–8.4) mg/dL
Calcium ionized at admission	4.8(4.8–5.2) mg/dL
Calcium ionized highest	5.2(4.8–5.6) mg/dL
Calcium ionized lowest	4.8(4.4–4.8) mg/dL
Phosphate at admission	1.5(1.2–2) mg/dL
Phosphate highest	1.9(1.5–2.3) mg/dL
Phosphate lowest	0.8(0.6–1.2) mg/dL
Magnesium at admission	1.94(1.7–7.2) mg/dL
Magnesium highest	2.2(1.9–2.4) mg/dL
Magnesium lowest	1.5(1.2–1.7) mg/dL
Uric acid at admission	7.1(4.2–10.3) mg/dL
Uric acid highest	8.2(5.7–11.6) mg/dL
Uric acid lowest	2.7(1.7–5.2) mg/dL

Table S2: Chemical and coagulation profile of patients with AKI diagnosis

**Parameter**	**Median (IQR)**
Albumin at admission	3.7(3.1–4.4) g/L
Albumin highest	3.9(3.4–4.4) g/L
Albumin lowest	2.8(2.1–3.4) g/L
AST enzyme at admission	64(32–203) IU/L
AST enzyme highest	133(40–448) IU/L
AST enzyme lowest	36(25–61) IU/L
ALT enzyme at admission	39(20–121) IU/L
ALT enzyme highest	63(26–235) IU/L
ALT enzyme lowest	25(17–51) IU/L
Prothrombin time at admission	12(11–15) Sec
Prothrombin time highest	14(11–19) Sec
Prothrombin time lowest	11(10–12) Sec
INR at admission	1.1(1–1.4)
INR highest	1.3(1–1.8)
INR lowest	1(0.9–1.1)
PTT at admission	29(25–40) Sec
PTT highest	34(28–58) Sec
PTT lowest	26(23–30) Sec
D Dimers at admission	5.5(2.5–12)
D Dimers highest	9(2.8–25)
D Dimers lowest	4(1.4–7)
